# The Antibacterial Effect* In Vitro* of Honey Derived from Various Danish Flora

**DOI:** 10.1155/2018/7021713

**Published:** 2018-06-19

**Authors:** Reem Dina Matzen, Julie Zinck Leth-Espensen, Therese Jansson, Dennis Sandris Nielsen, Marianne N. Lund, Steen Matzen

**Affiliations:** ^1^Department of Plastic Surgery and Breast Surgery, Zealand University Hospital, Roskilde, Denmark; ^2^Department of Food Science, Faculty of Science, University of Copenhagen, Denmark; ^3^Department of Biomedical Sciences, Faculty of Health and Medical Sciences, University of Copenhagen, Denmark

## Abstract

The mechanism behind the biologic actions of honey as a wound remedy has been intensively studied; however, there is no published data regarding any antibacterial effect of honey derived from Danish flora. We surveyed 11 honeys of various Danish floral sources for their antibacterial activity and compared them to a culinary processed commercial honey (Jakobsens) and a raw and a medical grade Manuka (*Leptospermum scoparium*) honey using the agar-well diffusion method. We tested the effect on three gram-positive bacteria (two strains of* Staphylococcus aureus* and one strain of* Staphylococcus epidermidis*) and two gram-negative bacteria (*Pseudomonas aeruginosa* and* Escherichia coli*). All samples, except the commercial honey, exhibited antibacterial activity, and samples derived from Water Mint (*Mentha aquatica),* Organic 2 (mixed organic flora), and Linden (*Tilia cordata*) honey had consistent effects on all bacteria tested and showed greater effect than medical grade and raw Manuka (*L. scoparium*) honey. The content of methylglyoxal was low in the Danish honey (< 2 *μ*g/mL) and significantly (p<0.05) higher in both the raw and the medical grade Manuka (*L. scoparium*) honey, where the concentrations were, respectively, 6.29 *μ*g/mL and 54.33 *μ*g/mL. The antibacterial effect of Danish honeys was mostly due to hydrogen peroxide. We conclude that honeys derived from Danish flora possess antibacterial effect, probably by a hurdle effect of viscosity, osmolality, acidity, bioactive peptides, and most importantly the content of hydrogen peroxide. These findings indicate that honeys of various Danish floral sources may have clinical potential, although further studies are necessary to elucidate this in order to determine whether the results of our* in vitro* experiments also apply to a clinical setting.

## 1. Introduction

Honey has drawn increasing attention as a remedy for wound treatment of different kinds, mainly due to a verified antibacterial activity [1]. Antibiotic resistance and chronic wound infections have increased the interest in antimicrobial treatments, including honey-based wound care products, and these have been registered with medical regulatory authorities as wound care agents in many countries, among others, the European Union, USA, and New Zealand. These products are mainly based on Manuka (*Leptospermum scoparium*) honey from New Zealand.

Honey is a collection of nectar and consists of sugar (75–79%), water (20%), proteins, vitamins, minerals, and antioxidants [2]. The mechanisms of action of honey have been studied intensively, and it is acknowledged that it exerts a wound healing effect through a series of physical and bioactive properties [1]. The antibacterial activity of honey can be attributed to the natural occurrence of the enzymatic production of hydrogen peroxide (H_2_O_2_) and a varied presence of phytochemical components such as methylglyoxal (MGO) [3, 4]. The concentration of H_2_O_2_ in honey is low while still having a disinfectant and tissue debridement effect without being cytotoxic and causing tissue damage [5]. Furthermore, honey has a low pH, high osmolality, and viscous properties, which inhibits the growth of microorganisms [6].

Honey of different geographical and floral origins may possess differences in antibacterial properties, which may be related to different chemical compositions of honeys [5, 6]. Honey derived from the* Leptospermum* species in New Zealand (Manuka) and Australia is characterized by a high antibacterial activity even in the presence of catalase, which is an enzyme destroying H_2_O_2_. This nonperoxide activity is attributed to the high concentration of MGO, which is derived from dihydroxyacetone, present in large amount in the nectar from* Leptospermum* species [5, 7]. But other factors, such as phytochemical substances and polypeptides like bee-defensin-1, may contribute to the overall biological effects of honey as well [8, 9].

The raw Danish honeys which are sold via farmer's markets or local shops are characterized by not being heated in the process of production. This is mainly due to the fact that the raw Danish honey submerges from local beekeepers that process the honey immediately in glasses ready for sale, unlike commercial honeys, which are heated in order to liquefy after being stored. This immediate processing without heating preserves the natural enzymatic properties of the honey, but there is also a small risk of bacterial contamination. In order to not jeopardize the antibacterial effect of honey and to eradicate microorganisms, such as* Clostridium botulinum* spores which are sometimes found in honey, medical grade honey should be sterilized by gamma-irradiation, and not by heating which destroys the enzyme glucose oxidase [10, 11].

Interestingly, there is no published data regarding any antibacterial effect of honey derived from Danish flora, despite a large tradition for production and sale of raw honey in Denmark and the obvious interest in developing a local, biological, and therapeutically useful remedy for wound care.

The aim of this study was to examine the biological activities of Danish honey of different floral sources, determine the antibacterial activity of the various types* in vitro*, and correlate this with the presence of H_2_O_2_ and MGO. Furthermore, we analyzed a commercial (heated) honey and a medical grade Manuka (*L. scoparium*) honey as well as a raw Manuka (*L. scoparium*) honey from New Zealand for comparison.

## 2. Material and Methods

### 2.1. Honey Samples

Honey samples of different floral sources (**Table 1**) were collected from local beekeepers between July and August 2016. In addition, one sample was a commercial culinary processed honey from a Danish manufacturer Jakobsens and consisted of a blend of acacia honeys originating from different areas in Eastern Europe. Also, a raw Manuka (*L. scoparium*) honey was obtained from a local producer from New Zealand and a commercial medical grade Manuka (*L. scoparium*) honey (*“Activon”, Advancis Medical*) was included. All samples were stored in sterilized containers in the dark at room temperature (20-22°C). All tests were performed blinded, and labels were given after the experimental work and statistical analysis were completed. The source of floral identity was provided by the beekeepers based on availability of different sources for the nectar at the time of collection, the location of the apiary, and the organoleptic properties of the honey. The samples were diluted for handling by adding sterile Milli-Q water at 37°C to reach the desired dilutions. Throughout the antibacterial experiments, a solution of 75% honey was used, unless otherwise stated.

### 2.2. Pathogens

The pathogens (Culture collection of Food Microbiology and Fermentation, Department of Food Science at University of Copenhagen, Denmark) used for this study included

- *Staphylococcus aureus* CCUG 1800;
- *Staphylococcus aureus* 1094-7;
- *Staphylococcus epidermidis* CCUG 39508;
- *Pseudomonas aeruginosa* SKN 1317;
- *Escherichia coli* K 12.

### 2.3. Procedure and Measurement

For testing the antibacterial effects of the honey samples the agar-well diffusion method was applied using Brain Heart Infusion (BHI) broth (CM0113) and BHI Agar (CM1136) as medium, prepared according to the instructions of the manufacturer (OXOID Ltd. Basingstoke, Hampshire, England).

The BHI agar was heated in microwave, then cooled to 50°C, and transferred into sterile tubes of 30 mL agar and 100 *μ*L of the tested pathogen (propagated in BHI broth overnight). The agar was stirred before pouring into sterile Petri-dishes and kept to solidify at room temperature for 30 min.

Wells (5 mm) were cut into the agar-dishes and 50 *μ*L of the honey samples was pipetted into each well. Milli-Q water served as reference. The dishes were incubated at 37°C for 48 h and the diameters of the growth inhibition zones were measured in centimeters to the nearest 0.05 cm.

To identify the primary antibacterial substance of the honey, following tests were performed: osmotic stress, dilution of the samples, thermal sensitivity, the effect of MGO, and enzyme sensitivity. All experiments were performed with duplicate samples of the honey, unless otherwise stated.

### 2.4. Osmotic Effect

A sample of 75% sucrose (w/w), corresponding to the amount of sugar in honey, was made by diluting 7.5 g sucrose in 2.5 g sterile Milli-Q water. A sample of 15% sucrose (w/w), corresponding to the amount of sugar in 20% honey samples, was made by diluting 1.5 g sucrose into 8.5 g sterile Milli-Q water. 50 *μ*l of the pure sugar samples was placed in the agar wells and procedure followed as described above.

### 2.5. Testing Thermal Sensitivity

500 *μ*L of each sample was added to Eppendorf tubes and heated in either a 60°C water bath or in a pot with boiling water (100°C) for 30 min. before testing.

### 2.6. Testing the Effect of Methylglyoxal

Two dilutions of 40% methylglyoxal in water solution (CAS 78-98-8, SIGMA-ALDRICH) were prepared. For a 0.02% concentration (200 *μ*g/mL), 10 *μ*l methylglyoxal was added to 19.99 mL sterilized water. For a 0.04% concentration (400 *μ*g/mL), 20 *μ*l methylglyoxal was added to 19.98 mL sterilized water.

### 2.7. Testing Enzyme Sensitivity

The honey samples were treated with two different enzymes, proteinase-K and catalase, to investigate the significance of a possible bioactive polypeptide and H_2_O_2_. 500 *μ*l of each honey sample (75% honey) was pipetted into two Eppendorf tubes. One sample was added 50 *μ*l of a 10 mg/ml proteinase-K solution K (CAS No. 39450-01-6, SIGMA-ALDRICH) for a 1 mg/mL solution. The other 500 *μ*l of each honey sample was added 10 *μ*l of a 50 mg/ml catalase solution (CAS No. 9001-05-2, SIGMA-ALDRICH) for a 1 mg/mL solution.

All samples were incubated at 37°C for 2 hours before being filled in the wells and tested as described previously. The experiments were only carried out on four out of the five pathogens mentioned in Section 2.2, thereby excluding* Pseudomonas aeruginosa* SKN 1317, due to no remarkably inhibiting activity in the previous experiments.

### 2.8. pH Measurement

pH measurements were carried out using a calibrated PHM250 Ion Analyzer-Radiometer Analytical.

### 2.9. Determination of MGO Concentration in Honeys

The determination of dicarbonyls in honey was performed according to Adams [12]. Briefly, 2 mL of Milli-Q water was mixed with 0.6 g of honey. Subsequently, 1.5 mL of the diluted honey was mixed with 0.75 mL 2%* o*-phenyl diamine (OPD) (98%; Sigma, Steinheim, Germany) in phosphate buffer (0.5 M, pH 6.5; Merck, Darmstadt, Germany), in triplicate, and left over night to derivatize (19 hours). After the derivatization, the samples were filtered through 0.2 *μ*m filters and analyzed by a method based on ultrahigh performance liquid chromatography (UHPLC) described by Hellwig et al. [13]. In short, the samples were separated on a Prontosil 60 phenyl material (250 mm*∗*4.6 mm, 5 *μ*m), with a guard column (Knauer, Berlin Germany, 5*∗*4 mm) filled with the same material and an online filter (3 *μ*m). The injection volume was 50 *μ*L, flow rate was 0.7 mL/min, and the UV detection was 312 nm. Eluent A consisted of 0.075% acetic acid (Sigma, Steinheim, Germany), and eluent B was 80% methanol (Sigma, Steinheim, Germany) and 20% of eluent A. The gradient was as follows: 10% B to 50% B from 0 to 27 min. by a linear gradient, 50% B kept constant from 27 to 30 min., increase from 50% to 70% B from 30 to 34 min. by a linear gradient followed by an increase to 100% B to 44 min., which was kept constant from 44 to 48 min., and finally back to 10% B from 48 to 50 min. by a linear gradient. A standard curve was prepared with the quinoxaline of MGO (Sigma, Steinheim, Germany) in the range between 0.4 *μ*g/mL and 20 *μ*g/mL.

### 2.10. Statistical Analyses

As the distribution of data was assumed normal, the statistical analysis was carried out using Excel v15.26 and StatPlus 2016 v6.1.60 for t-tests and one-way ANOVA tests. In addition, measurements of least squares means (lsmeans) were applied on the data for pairwise comparison of the MGO data (alfa=0.05) by the software RStudio (RStudio Team (2015), version 0.99.446, RStudio: Integrated development for R. RStudio, Inc., Boston, MA).

## 3. Results

**Figure 1:**
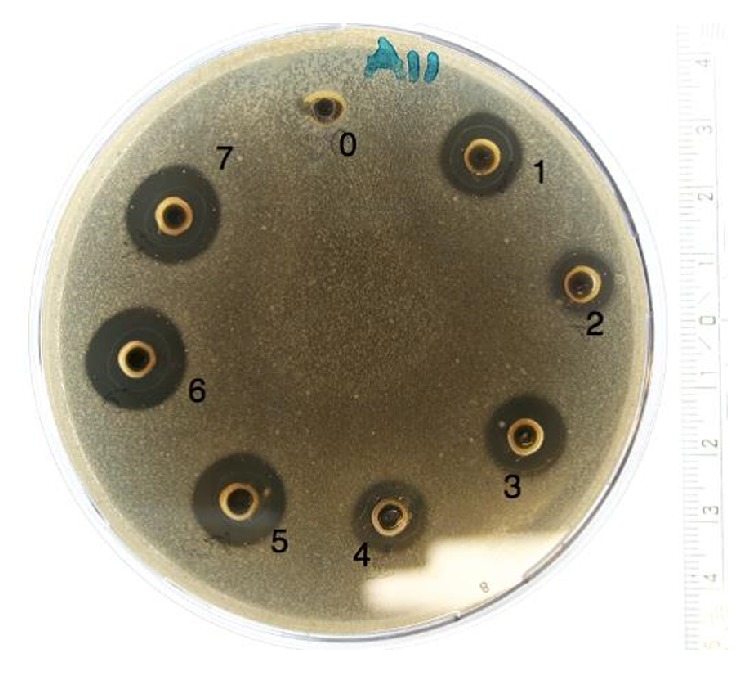
Photo of an agar-dish after agar-well diffusion assay. The dish is inoculated with* Staphylococcus aureus* (CCUG 1800) and honeys 1-7 are added in the wells. Milli-Q water serves as reference [0].

All the Danish honeys had antibacterial effect (p<0.05), and the honey samples Organic 2 (mixed organic flora), Water Mint (*Mentha aquatica*), and Linden (*Tilia cordata*) even possessed specific activity against* E. coli* and* P. aeruginosa*, while the medical grade Manuka (*L. Scoparium*) honey showed no activity at all towards these species. There were no significant differences (p>0.05) between the duplicate testing of all samples and all controls did not show any significant value throughout the various experiments. An example of the test results after agar diffusion method is presented in**Figure 1**.

**Figure 2:**
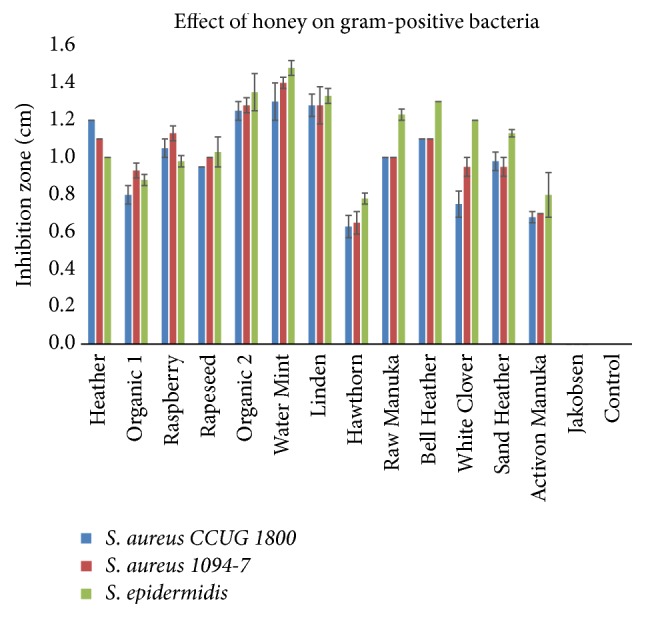
Effect of different floral sources of honey on growth of gram-positive bacteria (mean ± SD; n = 2).

**Figure 3:**
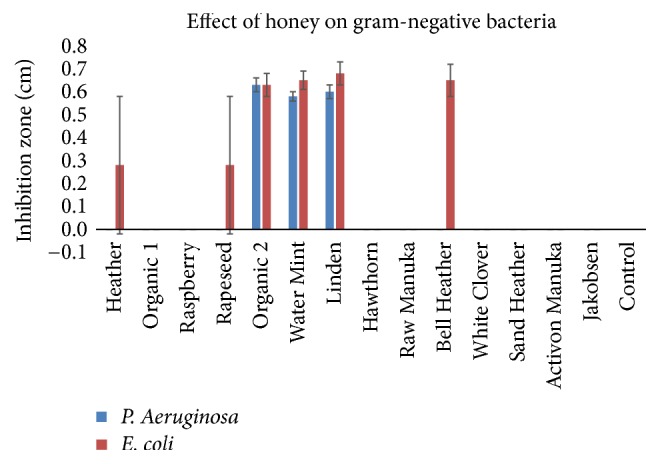
Effect of different floral sources of honey on growth of gram-negative bacteria (mean ± SD; n = 2).

The bar charts in** Figures 2 and 3** disclose the antibacterial effect of the honey samples. The Water Mint (*M. aquatica*), Linden (*T. cordata*), and Organic 2 (mixed organic flora) were able to inhibit all of the tested pathogens, showed the greatest inhibition zones and had a significant (p<0.05) effect on the gram-negative pathogens. The antibacterial effect of the honeys was greatest on the three gram-positive pathogens as compared to the gram-negative pathogens. The two honeys Hawthorn (*Crataegus monogyna*) and Activon Manuka (*L. scoparium*) showed the least inhibitory effect on the three gram-positive* Staphylococci *and were not able to inhibit the two gram-negative bacteria* P. aeruginosa *and* E. coli*. The commercial honey, Jakobsens, had no antibacterial effect in any of the tests.

### 3.1. Osmotic Effect

The pure sugar samples (75% and 15% sucrose) did not show any inhibition on the five selected pathogens.

### 3.2. Thermal Sensitivity

**Figure 4:**
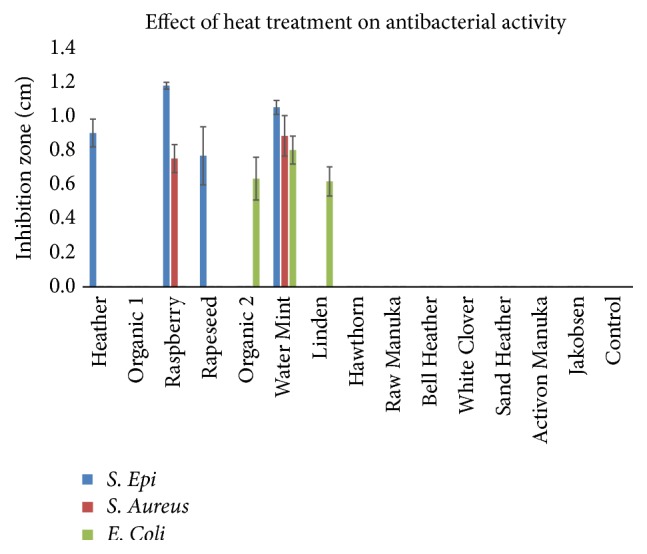
Antimicrobial effect of honey samples after heat treatment at 60°C/30 min. Some samples still showed antibacterial activity. These samples include Heather, Raspberry, Rapeseed, Organic 2, Water Mint, and Linden (mean ± SD; n = 2).

The heat treatment of the honey samples revealed a reduction in the inhibitory effect on the tested pathogens. The heat treatment of the honey samples at 100°C in 30 min. inhibited all antimicrobial effect in all honey samples; however, honey samples Heather (*Calluna vulgaris*), Raspberry (*Rubus odoratus*), Rapeseed (*Brassica napus*), Organic 2 (mixed organic flora), Water Mint (*M. aquatica),* and Linden (*T. cordata*) were able to inhibit microbial growth of some of the pathogens after heat treatment at 60°C (**Figure 4**).

### 3.3. Effect of MGO

Pure MGO solutions containing 200 *μ*g/mL or 400 *μ*g/mL showed an inhibitory effect on four out of the five tested pathogens, as* P. aeruginosa *samples were excluded from the study due to contamination. At a concentration of 400 *μ*g/mL MGO, an inhibitory effect was seen on all four tested pathogens and significantly (p<0.05) higher compared to the 200 *μ*g/mL MGO in three of the tested pathogens (*S. aureus* (1094-7),* S. epidermidis*, and* E. coli*).

### 3.4. Effect of Proteinase-K

All samples showed varying inhibitory effects on the different bacteria. For* S. aureus *(1094-7), honey samples Organic 1 (mixed organic flora), Rapeseed (*B. napus*), Water Mint (*M. aquatica*), Hawthorn (*C. monogyna*), and Bell Heather (*Erica tetralix*) had significantly (p<0.05) reduced activity after proteinase-K treatment. For* S. epidermidis, *comparable results were observed with Raspberry (*R. odoratus*), Hawthorn (*C. monogyna*), and Bell Heater (*E. tetralix*) (p<0.05). For* E. coli*, proteinase-K treatment resulted in a significant (p<0.05) decrease in nine of the 13 honey samples, and in six of the samples the inhibitory effect was lost completely (**Table 2**).

### 3.5. Effect of Catalase

Treatment with catalase abolished the antimicrobial effect of all the Danish honey samples, while the Activon Manuka (*L. scoparium*) maintained a significant antibacterial effect on S.* aureus* (1094-7) and* epidermidis* (p<0.05) (**Table 3**).

### 3.6. pH in Honey

**Figure 5:**
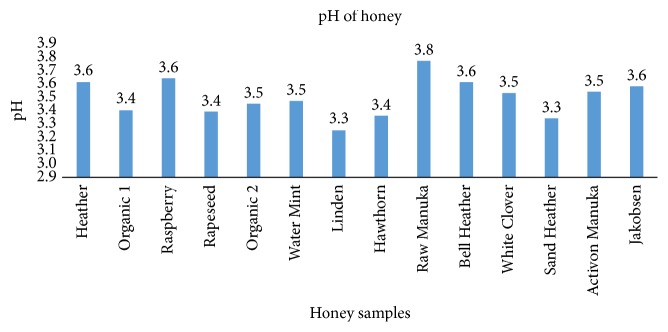
pH values of the 14 tested honey samples. The measurements are performed on a solution of 20% honey (*n* = 1).

The 14 honey samples had a pH varying between 3.25 and 3.77 (mean: 3.49 and standard variation: 0.14) (**Figure 5**).

### 3.7. Methylglyoxal in Honey Samples

**Figure 6:**
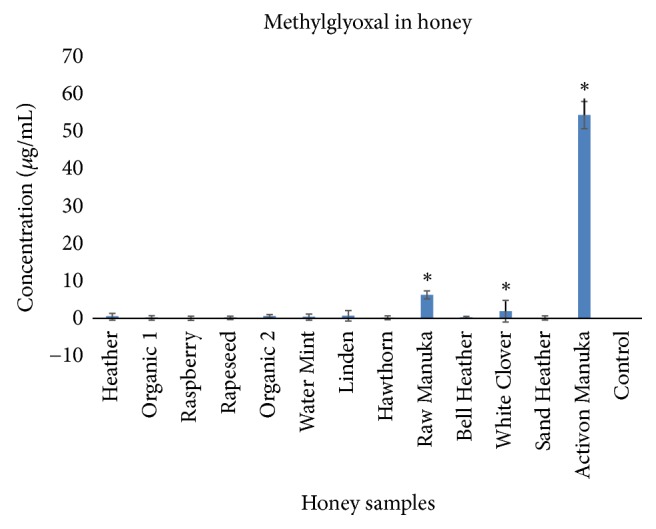
Concentration of methylglyoxal in 13 of the tested honey samples (mean ± SD, n=3). Symbol (*∗*) denotes statistical difference (p<0.05).

The concentrations of MGO in Activon Manuka (*L. scoparium*) and in raw Manuka honey (*L. scoparium*) were, respectively, 54.33 *μ*g/mL and 6.29 *μ*g/mL, and these concentrations were significantly higher than the other honey samples (**Figure 6**).

## 4. Discussion

This is the first study demonstrating that honey derived from Danish flora exhibit antimicrobial effects. This biological effect was for some honeys similar to or higher than the antibacterial effect of Manuka (*L. scoparium*) honey, especially regarding the inhibition of gram-negative microorganisms. The mechanism of action is mainly due to the content of H_2_O_2_ present in the Danish honey. The content of MGO is low in the Danish honeys compared to the medical grade Manuka (*L. scoparium*) honey, and the influence of this phytochemical substance on the antimicrobial effect of Danish honeys is probably of minor significance.

Clear differences could be observed in the antibacterial effect of the floral sources of honey with the Water Mint (*M. aquatica*), Linden (*T. cordata*), and Organic 2 (mixed organic flora) having the most consistent antibacterial effects on all tested pathogens. Also, the commercial culinary processed honey does not show any inhibition of the pathogens. This result, in conjunction to the results from the pure sugar samples, indicates that the antimicrobial effect is not simply conditional to the sugar content in the honey.

The gram-positive target strains were the most susceptible to honey whereas the gram-negative microbes were less sensitive to all honey samples including Manuka (*L. scoparium*), which is in accordance with previous observations [9, 14]. The difference in susceptibility to honey and other antibacterial agents between gram-positive and gram-negative microbes may be due to the composition of the cell wall. Gram-positive bacteria do not have an outer membrane protecting the peptidoglycan layer in contrast to gram-negative bacteria making it easier for antimicrobial agents to penetrate and cause damage [15].

The production of H_2_O_2_ by the presence of the enzyme glucose oxidase in honey is considered to be an important factor for the overall antibacterial effect of honeys [16]. But the concentration does not reach levels that are considered cytotoxic [17], which could be of relevance in case of applying honey as a remedy for wound care [18]. In the present study it was observed that treating the honey samples with catalase, an enzyme inhibiting glucose oxidase and thereby the production of H_2_O_2_, significantly reduced the antibacterial effect of all the Danish honeys. However, the antibacterial effect of the Manuka (*L. scoparium*) honey on some of the gram-positive strains was unaffected. This finding is in accordance with previous observations that more important factors than the level of H_2_O_2_ accounts for the antibacterial effect of this sort of honey [1, 19]. It was confirmed that MGO, at least in the higher concentration (400 *μ*g/mL), had an antibacterial effect, and together with the finding of a high level of MGO in the Manuka (*L. scoparium*) honey samples, this substance can be accountable for most of the antibacterial effect of this honey as reported previously [19]. In contrast, the Danish honeys had very low MGO levels, which is why this substance has little or no significance to the antibacterial effects of these particular honey types. On the other hand, studies have indicated that honeys with H_2_O_2_-dependent activity may be more broad spectrum and therapeutically useful as antifungal agents than Manuka honey, because they were found to be more effective than Manuka honey at inhibiting dermatophyte fungi [20] and species of the yeast* Candida* [21].

Besides the production and content of H_2_O_2_ or MGO, other properties of honey may contribute to the overall effects on bacteria. The low water content of honey and high osmolality and viscosity, acidic pH, and presence of leptosin and polypeptides like bee-defensin-1 have all been found to contribute to the reduced bacterial growth [22]. The low pH of honey did not seem to be an important factor in the present study. High osmolality is presumed to add to the antimicrobial effect of honey. However, from our experiments, high concentrations of glucose alone did not show any inhibition of bacterial growth. Furthermore, the heated preprocessed commercial honey had no antibacterial effect at all, despite a presumed high level of sugar. Other studies also indicate that solutions of glucose has less antimicrobial effect than honey [23], and the sugar-induced osmolality in honey is merely regarded as contributing to an unfavorable environment for pathogens, rather than being a primary inhibiting factor on bacterial growth by itself.

For further characterization of the mechanisms behind the antibacterial effect observed in the Danish honeys, the involvement of bioactive peptides was investigated by adding proteinase-K to the different honey samples. Proteinase-K was expected to cleave the proteins and thereby inactivate bioactive peptides such as defensin-1 [5]. Defensins are antibacterial peptides created to protect the host cells from invasion and infection by pathogens [24]. We found overall smaller inhibition zones on the agar plates with some differences between pathogens tested and the honey types after application of proteinase-K. It has previously been reported that there are differences in the presence of bee-defensin-1 in different sorts of honeys [9]. Our study shows a reduced effect on bacterial growth after adding a proteolytic enzyme and this is indicative of involvement of a biologic active peptide. However, further studies and other methods are necessary to elucidate if Danish honeys contain bioactive bee-defensin-1.

Throughout the experiments, the agar-well diffusion method was applied and used with a 75% solution of honey, for practical reasons, to determine the antibacterial effects of the honey samples. The 75% solution of honey had significant effect on the bacterial growth, while no effect was seen for the 20% solution of honey. This finding was also applied for the Manuka (*L. scoparium*) honey. It is well established that the agar-well diffusion method is suited for testing antibacterial effect, but if minimum inhibitory concentrations (MIC) are to be calculated, a more sensitive method like agar or broth dilution, where the honey is incorporated directly into the agar growth media must be used [25]. By this method the microbes are brought into direct contact with the testing inhibiting substance, and thereby not relying on the agent's ability to diffuse through the agar media [26].

Due to the physical characteristics of honey, it may contribute to a moist environment, which is beneficial for wound healing [27]. Furthermore, honey has been shown to stimulate the immune response and reduce inflammation, which in turn leads to an accelerated wound healing [1, 28, 29]. In addition, honey may also reduce the need for surgical wound debridement in selected cases [18, 30]. However, if honey is to be applied as a medical remedy for wound care it is necessary to process the honey for sterilization in order to eliminate a possible presence of pathogens or* C. botulinum* spores [11], why sterilization by gamma-irradiation should be performed [10]. No significant change in the antibacterial activity of honey was found caused by this method of sterilization of honey, neither in the honeys with H_2_O_2_-dependent activity or in the Manuka honeys [10]. Additionally, no significant changes were found in the physiochemical and mineral contents of honey resulting from sterilization by gamma-irradiation [31]. The process of heating honey will eliminate pathogens but also reduce the activity of H_2_O_2_ and other antibacterial substances. This is verified in the present study where the antibacterial activity was inhibited in most of the samples by heating the honey to 60°C.

## 5. Conclusion

This is the first study providing a substantial* in vitro* investigation of the antibacterial effect of honey derived from various Danish flora. We verified great variation in different floral sources with the Water Mint (*M. aquatica*), Linden (*T. cordata*), and Organic 2 (mixed organic flora) possessing the highest antibacterial activity on all the tested pathogens. These Danish honeys were comparable and even superior to commercial medical grade honey. The antibacterial effect was probably due to the activity of H_2_O_2_, though no direct measurements of the concentration of this substance was performed. Other studies have also been able to verify variation in antibacterial activity of honey depending on geographical location and floral source [26, 32].

Since the foraging of bees is not completely controllable and depends on the dominant floral source at the time of collection, it will be almost impossible to standardize a natural monofloral honey. Therefore, it is reasonable to assume that a specific floral source of honey also builds on a certain percentage of nonspecific nectar. However, while the antibacterial activity of honey might be a result of a hurdle effect of the honey's phytochemical characteristics, pH, viscosity, and content of H_2_O_2_, the mixture of different honey types might prove superior to a monofloral honey. Further studies are necessary to elucidate this hypothesis and to determine whether the results of our* in vitro* experiments also apply to a clinical setting.

## Figures and Tables

**Table 1:** Honey samples included in the study. The Danish samples were obtained by two local beekeepers from the Zealand Region. The Activon Manuka is a medical grade honey and was obtained from Advancis Medical. The raw Manuka was obtained from a local beekeeper in New Zealand.

Sample no.	Floral source Common name (Scientific name)
1	Heather (*Calluna vulgaris*)
2	Organic 1-mixed organic flora
3	Raspberry (*Rubus odoratus*)
4	Rapeseed (*Brassica napus*)
5	Organic 2-mixed organic flora
6	Water mint (*Mentha aquatica*)
7	Linden (*Tilia cordata*)
8	Hawthorn (*Crataegus monogyna*)
9	Raw Manuka (*Leptospermum scoparium*)
10	Bell Heather (*Erica tetralix*)
11	White clover (*Trifolium repens*)
12	Sand heather (*Hudsonia tomentosa*)
13	Activon Manuka (*Leptospermum scoparium*)
14	Jakobsens

**Table 2:** Mean zones of inhibition (mm) before (-) and after (+) treatment with the proteolytic enzyme proteinase-K on the 14 different honey samples.

Bacteria	1	2	3	4	5	6	7	8	9	10	11	12	13	14
*Staph. aureus*														
*CCUG 1800*														
-	10.5 ± 0.7	3.5 ± 0.4	9.5 ± 0.7	9.0 ± 1.4	13.8 ± 1.1	15.0 ± 2.1	12.5 ± 0.4	6.3 ± 0.4	8.8 ± 0.4	11.2 ± 1.1	9.3 ± 0.4	10.8 ± 1.8	0.0 ± 0.0	0.0 ± 0.0
+	7.5 ± 0.7	0.0 ± 0.0	8.0 ± 0.7	7.3 ± 0.4	11.0 ± 1.4	12.3 ± 1.1	10.0 ± 0.7	0.0 ± 0.0^*∗*^	7.7 ± 0.4	8.5 ± 0.7	8.0 ± 1.4	9.0 ± 0.7	0.0 ± 0.0	0.0 ± 0.0

*Staph. aureus*														
*(1094-7)*														
-	9.5 ± 0.7	7.7 ± 0.4	10.0 ± 0.4	7.7 ± 0.4	-	-	12.0 ± 0.0	6.8 ± 0.4	10.8 ± 1.1	11.3 ± 0.4	-	-	-	-
+	8.5 ± 0.7	0.0 ± 0.0^*∗*^	8.5 ± 0.4	0.0 ± 0.0^*∗*^	-	-	11.0 ± 0.1	0.0 ± 0.0^*∗*^	8.3 ± 0.4	8.3 ± 0.4^*∗*^	-	-	-	-

*Staph. epi*														
(CCUG 39508)														
-	10.5 ± 0.7	3.8 ± 5.3	10.3 ± 0.4	9.0 ± 1.4	14.3 ± 1.1	15.0 ± 1.4	11.5 ± 0.7	6.0 ± 0.0	9.9 ± 0.7	10.0 ± 0.0	10.0 ± 1.4	9.7 ± 1.1	7.3 ± 0.4	0.0 ± 0.0
+	8.0 ± 0.7	0.0 ± 0.0	7.8 ± 0.4^*∗*^	3.8 ± 5.3	11.3 ± 1.1	11.0 ± 1.1	9.5 ± 0.0	0.0 ± 0.0^*∗*^	7.8 ± 0.4	7.5 ± 0.7^*∗*^	7.0 ± 0.0	7.7 ± 0.7	6.5 ± 0.0	0.0 ± 0.0

*E. coli*														
*(K 12) *														
-	8.3 ± 0.4	3.3 ± 4.6	7.3 ± 0.4	7.3 ± 0.0	10.5 ± 8.0	11.3 ± 0.4	9.5 ± 0.7	6.0 ± 0.3	7.8 ± 0.4	8.0 ± 0.2	7.5 ± 0.2	8.0 ± 0.5	6.3 ± 0.4	-
+	7.0 ± 0.0^*∗*^	0.0 ± 0.0	0.0 ± 0.0^*∗*^	0.0 ± 0.0^*∗*^	8.0 ± 0.0^*∗*^	8.5 ± 0.0^*∗*^	7.8 ± 0.4	0.0 ± 0.0^*∗*^	7.0 ± 0.0^*∗*^	7.0 ± 0.0^*∗*^	0.0 ± 0.0^*∗*^	7.0 ± .4^*∗*^	3.0 ± 4.2	-

The antibacterial effect of different honey types on four different bacteria was assessed by the agar well diffusion method on duplicate samples and the mean ± SD are presented.

Symbol (-) indicates insufficient samples (n=1 or 0).

Symbol (*∗*) denotes statistically significance (p< 0.05; enzyme treatment versus no treatment).

**Table 3:** Mean zones of inhibition (mm) before (-) and after (+) treatment with the enzyme catalase on the 14 different honey samples.

Bacteria	1	2	3	4	5	6	7	8	9	10	11	12	13	14
*Staph. aureus*														
*CCUG 1800*														
-	8.0 ± 0.7	6.3 ± 0.4	7.5 ± 0.0	7.5 ± 0.0	7.0 ± 0.0	11.0 ± 2.1	8.8 ± 0.4	6.0 ± 0.4	7.8 ± 0.4	8.8 ± 0.4	-	-	-	0.0 ± 0.0
+	0.0 ± 0.0^*∗*^	0.0 ± 0.0^*∗*^	0.0 ± 0.0^*∗*^	0.0 ± 0.0^*∗*^	0.0 ± 0.0^*∗*^	0.0 ± 0.0^*∗*^	0.0 ± 0.0^*∗*^	0.0 ± 0.0^*∗*^	0.0 ± 0.0^*∗*^	0.0 ± 0.0^*∗*^	00 ± 0.0	0.0 ± 0.0	-	0.0 ± 0.0

*Staph. aureus*														
*(1094-7)*														
-	9.8 ± 2.5	7.8 ± 0.4	10.5 ± 0.7	787 ± 1.8	13.3 ± 1.1	13.5 ± 0.7	12.0 ± 0.0	6.0 ± 0.0	10.0 ± 0.0	10.0 ± 0.0	9.3 ± 0.4	10.5 ± 0.7	7.5 ± 0.1	0.0 ± 0.0
+	0.0 ± 0.0^*∗*^	0.0 ± 0.0^*∗*^	0.0 ± 0.0^*∗*^	0.0 ± 0.0^*∗*^	0.0 ± 0.0^*∗*^	0.0 ± 0.0^*∗*^	0.0 ± 0.0^*∗*^	0.0 ± 0.0^*∗*^	0.0 ± 0.0^*∗*^	0.0 ± 0.0^*∗*^	0.0 ± 0.0^*∗*^	0.0 ± 0.0^*∗*^	6.5 ± 0.1^*∗*^	0.0 ± 0.0

*Staph. epi*														
(CCUG 39508)														
-	7.5 ± 0.0	0.0 ± 0.0	7.0 ± 0.0	7.0 ± 0.0	8.0 ± 0.0	9.0 ± 0.7	7.3 ± 0.4	6.0 ± 0.0	7.8 ± 0.4	7.8 ± 0.4	7.5 ± 0.4	7.8 ± 0.4	6.5 ± 0.1	0.0 ± 0.0
+	0.0 ± 0.0^*∗*^	0.0 ± 0.0^*∗*^	0.0 ± 0.0^*∗*^	0.0 ± 0.0^*∗*^	0.0 ± 0.0^*∗*^	0.0 ± 0.0^*∗*^	0.0 ± 0.0^*∗*^	0.0 ± 0.0^*∗*^	0.0 ± 0.0^*∗*^	0.0 ± 0.0^*∗*^	0.0 ± 0.0^*∗*^	0.0 ± 0.0^*∗*^	6.0 ± 0.1	0.0 ± 0.0

*E. coli*														
*(K 12)*														
* -*	0.0 ± 0.0	0.0 ± 0.0	0.0 ± 0.0	0.0 ± 0.0	6.0 ± 0.0	6.8 ± 0.4	6.3 ± 0.4	0.0 ± 0.0	0.0 ± 0.0	6.0 ± 0.0	0.0 ± 0.0	0.0 ± 0.0	0.0 ± 0.0	0.0 ± 0.0
+	0.0 ± 0.0	0.0 ± 0.0	0.0 ± 0.0	0.0 ± 0.0	0.0 ± 0.0^*∗*^	0.0 ± 0.0^*∗*^	0.0 ± 0.0^*∗*^	0.0 ± 0.0	0.0 ± 0.0	0.0 ± 0.0^*∗*^	0.0 ± 0.0	0.0 ± 0.0	0.0 ± 0.0	0.0 ± 0.0

The antibacterial effect of different honey types on four different bacteria was assessed by the agar well diffusion method on duplicate samples and the mean ± SD are presented.

Symbol (-) indicates insufficient samples (n=1 or 0).

Symbol (*∗*) denotes statistically significance (p< 0.05; enzyme treatment versus no treatment).

## Data Availability

The data used to support the findings of this study are available from the corresponding author upon request.
